# The burden of hand, foot, and mouth disease among children under different vaccination scenarios in China: a dynamic modelling study

**DOI:** 10.1186/s12879-021-06157-w

**Published:** 2021-07-05

**Authors:** Zhixi Liu, Jie Tian, Yue Wang, Yixuan Li, Jing Liu-Helmersson, Sharmistha Mishra, Abram L. Wagner, Yihan Lu, Weibing Wang

**Affiliations:** 1 School of Public Health, Fudan University, Shanghai, 200032 China; 2 Department of Epidemiology and Global Health, Faculty of Medicine, Umeå University, 90187 Umeå, Sweden; 3 Department of Medicine, Institute of Medical Sciences, and Institute of Health Policy, Management and Evaluation, University of Toronto, Toronto, Canada; 4 Center for Urban Health Solutions, Li Ka Shing Knowledge Institute, University of Toronto, Toronto, Canada; 5 Department of Epidemiology, University of Michigan, Ann Arbor, MI 48109 USA

**Keywords:** Hand, foot and mouth disease, SEIR model, Vaccine, Basic reproductive number, Pulse vaccination

## Abstract

**Background:**

Hand, foot, and mouth disease (HFMD) is a common illness in young children. A monovalent vaccine has been developed in China protecting against enterovirus-71, bivalent vaccines preventing HFMD caused by two viruses are under development.

**Objective:**

To predict and compare the incidence of HFMD under different vaccination scenarios in China.

**Methods:**

We developed a compartmental model to capture enterovirus transmission and the natural history of HFMD in children aged 0–5, and calibrated to reported cases in the same age-group from 2015 to 2018. We compared the following vaccination scenarios: different combinations of monovalent and bivalent vaccine; a program of constant vaccination to that of pulse vaccination prior to seasonal outbreaks.

**Results:**

We estimate 1,982,819, 2,258,846, 1,948,522 and 2,398,566 cases from 2015 to 2018. Increased coverage of monovalent vaccine from 0 to 80% is predicted to decrease the cases by 797,262 (49.1%). Use of bivalent vaccine at an 80% coverage level would decrease the cases by 828,560. Use of a 2.0× pulse vaccination for the bivalent vaccine in addition to 80% coverage would reduce cases by over one million. The estimated *R*_*0*_ for HFMD in 2015–2018 was 1.08, 1.10, 1.35 and 1.17.

**Conclusions:**

Our results point to the benefit of bivalent vaccine and using a pulse vaccination in specific months over routine vaccination. Other ways to control HFMD include isolation of patients in the early stage of dissemination, more frequent hand-washing and ventilation, and better treatment options for patients.

**Supplementary Information:**

The online version contains supplementary material available at 10.1186/s12879-021-06157-w.

## Introduction

Hand, foot, and mouth disease (HFMD) is a common infectious disease mainly caused by various enteroviruses. HFMD usually affects children under age of five, with a incidence rate of approximately 2400 cases per 100,000 in 2018 in China [1, 2], and occurs more often in children under three [3]. After 2007, the HFMD epidemic was in an uptrend in China accompanied by serious outbreaks [4]. Although HFMD is usually self-limiting, it can result in complications associated with the central nervous system or death once progressing to severe cases [5, 6]. There are more than 20 types of enterovirus leading to HFMD and Enterovirus 71 (EV71) and Coxsackie virus A16 (CV-A16) are the most commonly reported [3]. EV71 accounts for 70% severe HFMD cases and 90% HFMD-related deaths in mainland China [7].

Mathematical models of enterovirus transmission are increasingly being used to design and inform public health interventions to reduce the spread of HFMD. For example, Chuo et al. developed a deterministic susceptible-infectious-recovered (SIR) model to predict the number of infected people during an HFMD occurred in Sarawak, Malaysia and found that the disease spread quite rapidly and the parameter that may be able to control the number of susceptible persons [8]. Yang et al. used a SEIR model, adding a compartment to represent the exposed people (E) to capture the incubation period of HFMD and showed the spread trend of HFMD in China, and the basic reproduction number indicated that it was an outbreak [9]. By adding in a quarantined compartment (Q) in a SEIQR model, Liu simulated HFMD transmission and found that quarantine is the optimal strategy for HFMD protection [10].

EV71 vaccine is a new vaccine recently developed in China. The Vero cell-based EV71 inactivated vaccine has been shown to induce immune responses to EV71 among infants and young children between 6 and 59 months of age [11]. By March 2013, this inactivated vaccines had completed phase I-III clinical trials in Mainland China, showing good safety and greater than 95% protection against HFMD caused by EV71 [11–13]. Three manufactures in mainland China have recently completed phase III trials for EV71 vaccines and have been approved by China Food and Drug Administration in December 2015 [14]. In total, three monovalent EV71 vaccines have been licensed while bivalent EV71/CA16 vaccines are also under development and have been shown to induce protective immunity against both EV71 and CVA16 in vivo and in mice [15]. Further empirical studies are needed to assess the safety and effectiveness of these different vaccines in preventing HFMD.

As empiric data emerge on the safety and individual-level effectiveness of these vaccines, what remains unknown is their potential population-level impact on outbreaks and incidence of HFMD. Existing and future EV-71 and CV-A16 vaccines could differentially affect the incidence of HFMD. Thus, the aim of our study is to compare the predicted incidence of HFMD under different vaccination scenarios in China.

## Methods

We developed and analyzed a deterministic, compartmental, mathematical model of enterovirus transmission to reproduce the observed HFMD epidemic in China from 2015 to 2018. The modelled population includes one age-group to reflect all children from age zero to 5 years of age.

### Data sources

#### Literature review

We conducted a comprehensive review of peer-reviewed and grey literature including government reports to obtain the following model parameters: transmission rate, duration of infectiousness, serotypes distribution, and vaccine efficacy.

#### National HFMD surveillance dataset

HFMD refer to acute infectious diseases caused by enteroviruses such as human enterovirus 71 (EV-A71) and Coxsackie virus A group 16 (CV-A16), which are more common in preschool children. Severe cases are mostly caused by EV-A71 infection. The diagnostic criteria refer to the People’s Republic of China Hygiene Industry Standard for Hand, Foot and Mouth Disease Diagnosis (WS 588–2018). We obtained the number of reported cases of HFMD per month from 2015 to 2018 in China from the official infectious disease report from Chinese Centre for Disease Control and Prevention (Table S1) [2]. Since January 1, 2008, all probable and laboratory-confirmed HFMD cases have been reported to Chinese Centre for Disease Control and Prevention and by May 2, 2008, HFMD was a mandatory notifiable disease (III).

#### Demographic data

We obtained national-level estimates of the total number of children under 5, the birth rate, and the under-5 mortality rate from 2015 to 2018 from the National Bureau of Statistics and Provincial Bureau of Statistics [1].

### Model description

**Fig. 1:**
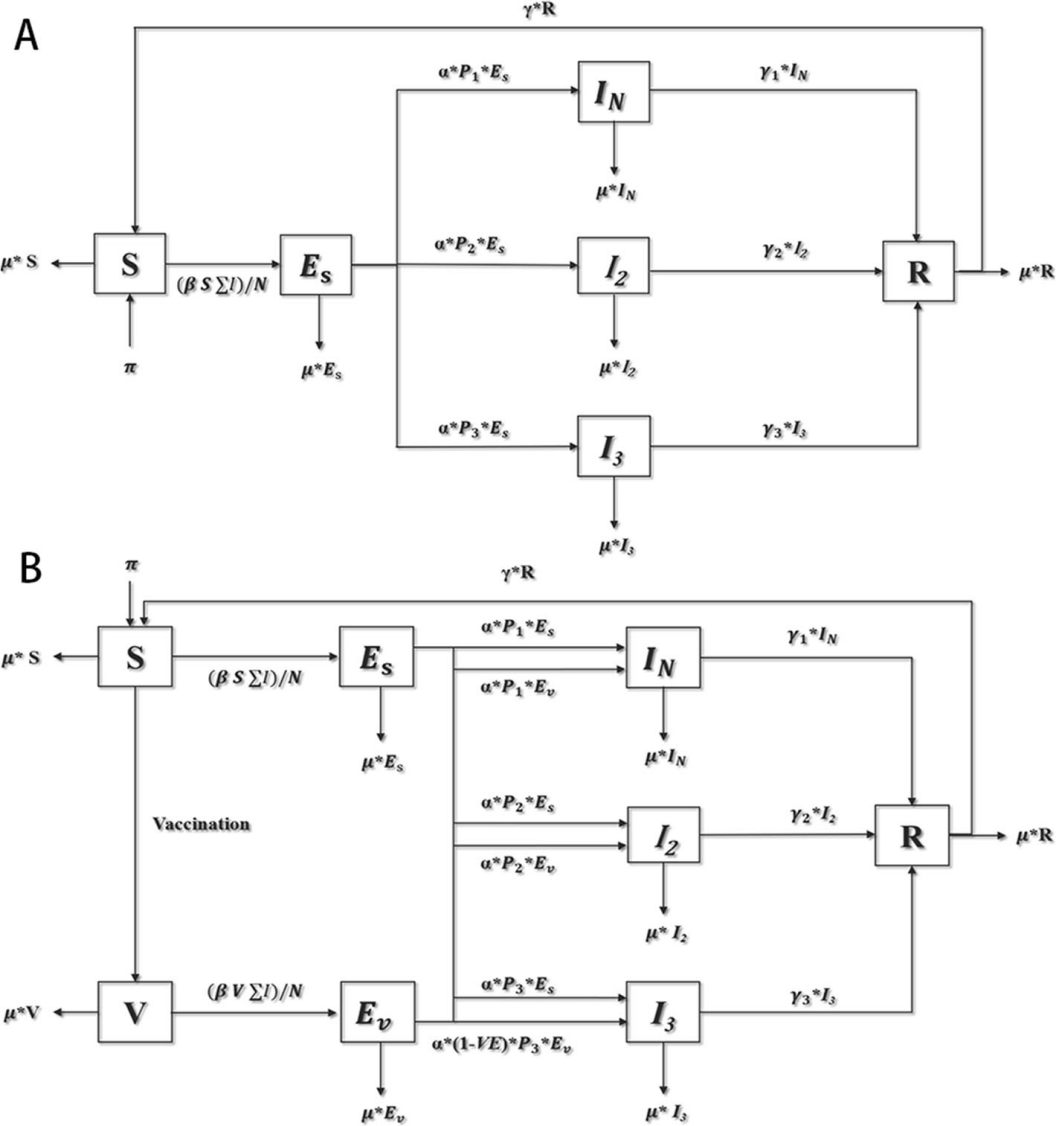
An extended SEIR model of Hand, foot and mouth diseases including 6 or 8 compartments. Figure 1**A** indicates the dynamic of HFMD transmission under no vaccination; Fig. 1**B** indicates the dynamic of HFMD transmission under vaccination

We developed an extended SEIR 6/8-compartment model to evaluate the spread and incidence of HFMD. The population is divided into six or eight compartments as shown in Fig. 1, based on the natural history of HFMD and intervention with vaccination. Specifically, the total population (N) was divided into four categories: susceptible to HFMD (S), exposed to HFMD-related pathogens (E), infectious (I) and recovered (R). With the exception of the recovered compartment, the other three compartments (S, E, and I) were further divided into 2 or 3 compartments based on vaccination, type of pathogens and hospitalization: vaccinated (V); exposed (E_s_); vaccinated and exposed (E_v_); infectious but not hospitalized including mildly ill patients and asymptomatic (I_N_); infectious hospitalized with pathogens other than EV71 or CVA16 (I_2_); infectious hospitalized with EV71-HFMD or CVA16-HFMD (I_3_) to capture the bivalent vaccine. Considering that the EV71 monovalent vaccine was actually introduced in 2017 in China, we should modify the meaning of I_2_ and I_3_ compartments in 2017 and 2018, where ${\mathrm{I}}_{\mathrm{h}}^{-\mathrm{e}}$ indicates people who are infectious with pathogens other than EV71 and ${\mathrm{I}}_{\mathrm{h}}^{\mathrm{e}}$ indicates people who are infectious with EV71-HFMD. The total population (N) is therefore given by *N* = *S* + *V* + *E*_*s*_ + *E*_*v*_ + *I*_*N*_ + *I*_2_ + *I*_3_ + *R*. Fig. 1 and Table 1 describe the model parameters.

**Table 1:** Model parameters, description, source and the basic reproduction number in China, 2015–2018

Parameters	Description	Source	2015	2016	2017	2018
S(0)*	Number of susceptible children under 5	Fixed [1]	95,920,000	96,490,000	98,290,000	94,386,870
V(0)	Vaccinated	Fixed [16]	0	0	0	5,110,000
E(0) in [500,5 ×10^4^]	Number of exposed under incubation periods	MSS [17]	3000	1800	2000	1200
I_N_(0) in [500,5 ×10^4^]	Infectious but not hospitalized	MSS [17]	1000	1200	1800	800
I_2_(0)	Infectious with pathogens other than EV71 or CVA16 / other than EV71	Calculated, 0.3/0.55 × reported cases in January/30**	577.5	795	1419	638
I_3_(0)	Infectious with EV71-HFMD or CVA16-HFMD / EV71-HFMD	Calculated, 0.7/0.45 × reported cases in January/30**	1347.5	1855	1161	522
R(0)	Recovered	Assumed	0	0	0	0
α	Progression rate	Assumed	1	1	1	1
β in [0.01,50]	Transmission rate, Contact frequency × Infection efficiency	MSS [17–19]	0.9387	0.7098	1.1188	0.8429
P_1_ in [0.01,1]	Proportion of I_N_	MSS [17]	0.9627	0.9663	0.8644	0.9333
P_2_	Proportion of I_2_	Calculated, 0.3/0.55 × (1-P_1_) **	0.0112	0.0101	0.0746	0.0367
P_3_	Proportion of I_3_	Calculated, 0.7/0.45 × (1-P_1_) **	0.0261	0.0236	0.0610	0.0300
γ_1_ in [0.1,1]	Recovery rate of I_N_	MSS[18]	0.8932	0.6441	1.0721	0.7573
γ_2_ in [0.1,1]	Recovery rate of I_2_	MSS[18]	0.8852	0.7127	0.2619	0.3612
γ_3_ in [0.1,1]	Recovery rate of I_3_	MSS[18]	0.4583	0.5497	0.4907	0.5264
γ in [0.0001,1]	Relapse rate	MSS*** [17]	0.0125	0.0136	0.0064	0.0139
π	Birth rate	Fixed [1]	0.01207/365	0.01295/365	0.01243/365	0.01094/365
μ	Death rate of children under 5	Fixed [1]	0.0107/365	0.0102/365	0.00905/365	0.00832/365
v	The number of vaccinated people per day	Fixed [16]	0	0	14,000	24,000
VE	Vaccine efficacy	Fixed [12]	–	–	95%	95%
R_0_	Basic reproductive number	Calculated	2.02	1.82	2.35	1.97

*(0) denotes the initial number of different compartments

**: The proportions of EV71, CV-A16 and other Enteroviruses are 45, 25 and 30% [20]. Therefore, the proportion of I_2_ (No EV71 or CVA16) and I_3_ (EV71 + CVA16) is 30 and 70% in 2015, 2016. While the proportion of I_2_ (No EV71) and I_3_ (EV71) is 55 and 45% in 2017 and 2018 due to the launch of EV71 vaccine

***: *MSS* minimum sum of square

The dynamics of HFMD is described in the following set of coupled ordinary differential equations to represent the dynamics of HFMD without vaccination.

1 $$ \left\{\begin{array}{c}\ \frac{dS}{dt}=\pi N+\gamma R-\frac{\beta S\sum I}{N}-\mu S\ \\ {}\ \frac{d{E}_s}{dt}=\frac{\beta S\sum I}{N}-\alpha \left({P}_1+{P}_2+{P}_3\right){E}_s-\mu {E}_s\ \\ {}\ \frac{d{I}_N}{dt}=\alpha {P}_1{E}_s-{\gamma}_1{I}_N-\mu {I}_N\ \\ {}\ \frac{d{I}_2}{dt}=\alpha {P}_2{E}_s-{\gamma}_2{I}_2-\mu {I}_2\\ {}\ \frac{d{I}_3}{dt}=\alpha {P}_3{E}_S-{\gamma}_3{I}_3-\mu {I}_3\ \\ {}\ \frac{dR}{dt}={\gamma}_1{I}_N+{\gamma}_2{I}_2+{\gamma}_3{I}_3-\gamma R-\mu R\end{array}\right. $$

The following set of eqs. (2) represent the dynamics of HFMD with the bivalent vaccine (protective against EV71 and CV-A16 simultaneously) and the monovalent vaccine (protective against EV71).

2 $$ \left\{\begin{array}{c}\frac{dS}{dt}=\pi N+\gamma R-\frac{\beta S\sum I}{N}-v-\mu S\\ {}\frac{dV}{dt}=v-\frac{\beta V\sum I}{N}-\mu V\\ {}\frac{d{E}_s}{dt}=\frac{\beta S\sum I}{N}-\alpha \left({P}_1+{P}_2+{P}_3\right){E}_s-\mu {E}_s\\ {}\frac{d{E}_v}{dt}=\frac{\beta V\sum I}{N}-\alpha \times \left[{P}_1+{P}_2+\left(1- VE\right)\times {P}_3\right]{E}_v-\mu {E}_v\ \\ {}\frac{d{I}_N}{dt}=\alpha {P}_1\left({E}_S+{E}_v\right)-{\gamma}_1{I}_N-\mu {I}_N\\ {}\frac{d{I}_2}{dt}=\alpha {P}_2\left({E}_S+{E}_v\right)-{\gamma}_2{I}_2-\mu {I}_2\ \\ {}\frac{d{I}_3}{dt}=\alpha {P}_3{E}_S+\left(1- VE\right)\alpha {P}_3{E}_v-{\gamma}_3{I}_3-\mu {I}_3\\ {}\frac{dR}{dt}={\gamma}_1{I}_N+{\gamma}_2{I}_2+{\gamma}_3{I}_3-\gamma R-\mu R\end{array}\right. $$

Formula (1) indicates the dynamic of HFMD under no vaccination. In formula (2), the vaccines both indicate the bivalent vaccine which can prevent children from being infected by EV71 and CV-A16 simultaneously and the monovalent vaccine which can only prevent children from being infected by EV71. We explicitly simulate the introduction of the EV71 vaccine in 2017 because the monovalent vaccine was introduced at that time in China (in December 2016) [14] .

### Model assumptions

We assumed that:

- All the newborns are susceptible.
- The epidemic was established by 2015 with the initial number of infectious [I_2_(0) + I_3_(0)] on January 1, 2015 equal to the number of reported cases in January 2015 divided by 31.
- We assumed that a vaccinated person has received the entire vaccination procedure (two doses of vaccination) and will be protected against EV71 or CV-A16 immediately after completing the full course of inoculation with EV71 or CV-A16 vaccine, and there is no cross-protection effect with other virus subtypes. The possibility of dominant virus subtypes of HFMD changing after vaccination is not considered.
- There is no HFMD-attributable mortality. According to the HFMD report of Chinese Centers for Disease Control, the number of deaths due to HFMD is extremely small, about one in ten thousand [2].
- Since the EV71 vaccines were launched in China in December 2016, the coverage of the vaccines is still very low. We assume that there is no impact of vaccination on the transmission rate β, and everyone was susceptible before and including 2016.
- We assumed contact patterns reflect mass action without heterogeneity by age nor mixing by age-groups. Thus, the contact rate is subsumed within the biological transmission probability β.
- We assumed I_N_ include asymptomatic

### Parameter estimation and model fitting

We estimate parameters by minimizing the sum of squares (MSS) [17] using the MATLAB R2018a (version 9.4) tool fminsearch [21] which is used to perform unconstrained nonlinear minimization. Posterior parameter values were obtained when the results of fminsearch converged.

3 $$ MSS=\sum {\left({\mathit{\log}}_2\left(1+ data\  per\  month\right)-{\mathit{\log}}_2\left(1+{I}_2\  and\ {I}_3\  on\  day1+ on\  day2+\dots + on\  day28, or29,30,31\right)\right)}^2 $$

Where I_2_ indicates the number of no EV71 or CVA16 HFMD $\Big({\mathrm{I}}_{\mathrm{h}}^{-\mathrm{ec}}$) / no EV71 HFMD $\left({\mathrm{I}}_{\mathrm{h}}^{-\mathrm{e}}\right)$, I_3_ indicates the number of EV71 or CVA16 HFMD $\left({\mathrm{I}}_{\mathrm{h}}^{\mathrm{ec}}\right)$ / only EV71- HFMD $\left({\mathrm{I}}_{\mathrm{h}}^{\mathrm{e}}\right)$.

In order to test the goodness of fit between our model and the observed data, we used a Chi-Square Test for the following hypotheses: Null hypothesis H_0_: The modeled results (I_2_ + I_3_) are equal to the observed number of HFMD cases shown in Table S1. Alternative hypothesis H_1_: The modeled results are not equal to the observed values shown in Table S1.

### Derivation of basic reproductive number (R_0_)

The basic reproductive number (*R*_*0*_) is of a great significance in Epidemiological studies. It is the expected number of secondary cases produced, in a completely susceptible population, after introducing a typical infective individual [22]. In this study, we can compute the basic reproduction number (*R*_*0*_) in 2015–2018 using the method of van den Driessche and assuming that each year represents an independent epidemic with a fully susceptible population on January 1 of each year [23]. See the Additional file 2 for the derivation process and results. In this model, *R*_*0*_ is related to transmission rate (β), progression rate (α), the proportion of I_N_, I_2_, I_3_ (P_1_, P_2_, P_3_), the recovery rate (γ_1_, γ_2_, γ_3_), the mortality rate (μ) and the vaccine efficacy (VE).

### Sensitivity analysis

Considering the formula of *R*_*0*_(Formula (2) in the Additional file 2, the expression of R_0_ in 2015 and 2016), the basic reproductive number is affected by β, α, P_1_, γ_1_, γ_2_ and γ_3_ while α is fixed to 1. Therefore, we take a derivation with respect to β, P_1_, γ_1_, γ_2_ and γ_3_, respectively. Derivation results are as follows:

4 $$ \left\{\begin{array}{c}\frac{d{R}_0}{d\beta}=\frac{1}{\alpha +\mu}\times \left(\frac{\alpha {P}_1}{\gamma_1+\mu }+\frac{0.3\times \alpha \left(1-{P}_1\right)}{\gamma_2+\mu }+\frac{0.7\times \alpha \left(1-{P}_1\right)}{\gamma_3+\mu}\right)\\ {}\frac{d{R}_0}{d{P}_1}=\frac{\beta \alpha}{\alpha +\mu}\times \left(\frac{1}{\gamma_1+\mu }-\frac{0.3}{\gamma_2+\mu }-\frac{0.7}{\gamma_3+\mu}\right)\ \\ {}\frac{d{R}_0}{d{\gamma}_1}=-\frac{\beta \alpha {P}_1}{\left(\alpha +\mu \right)\times {\left({\gamma}_1+\mu \right)}^2}\\ {}\frac{d{R}_0}{d{\gamma}_2}=-\frac{0.3\times \beta \alpha \left(1-{P}_1\right)}{\left(\alpha +\mu \right)\times {\left({\gamma}_2+\mu \right)}^2}\ \\ {}\frac{d{R}_0}{d{\gamma}_3}=-\frac{0.7\times \beta \alpha \left(1-{P}_1\right)}{\left(\alpha +\mu \right)\times {\left({\gamma}_3+\mu \right)}^2}\ \end{array}\right. $$

We further conducted a sensitivity analysis to measure the impact of these five parameters (β, P_1_, γ_1_, γ_2_ and γ_3_) on the number of EV71 or CV-A16 HFMD (I_3_) cases using Vensim PLP software (version 7.3.5, Ventana Systems, Inc.)

### Analyses of different vaccination type and coverage scenarios

We used the model to simulate the HFMD incidence of I_2_ and I_3_ under different vaccine coverage scenarios. To compare HFMD incidence under the monovalent EV-71 vaccine and bivalent vaccine coverage scenarios, we compared scenarios via a 5 × 6 vaccination matrix as follows: a) No EV-71 vaccination throughout the year; b) 10% EV-71 vaccination coverage (vaccination rate: 26,300 person/day); c) 30% EV-71 vaccination coverage (vaccination rate: 78,900 person/day); d) 50% EV-71 vaccination coverage (vaccination rate: 131,500 person/day); e) 80% EV-71 vaccination coverage (vaccination rate: 210,400 person/day); In each of the above cases, the coverage rates of bivalent vaccine coverage were 0, 10, 30, 50, 80 and 100%. We estimated the rate of vaccination for a certain vaccination coverage level to be the number of children < 5 divided by 365 and multiplied by the coverage level. We assumed vaccine protection rate was 95% and there was no loss of protective immunity following vaccination.

### Analyses of pulse vaccination

We then compared pulse vaccination strategies via increasing the vaccination rate in March, April and September (before the two peaks of HFMD incidence of a year) from 26,300 person/day (10% coverage rate), 78,900 person/day (30% coverage rate), 131,500 person/day (50% coverage rate) and 210,400 person/day (80% coverage rate) to 1.2 times, 1.4 times, 1.6 times, 1.8 times and 2.0 times, respectively. The annual vaccine coverage rates under different pulse vaccination strategies mentioned above should not be more than 100%. Therefore, we just introduced this strategy into 10, 30, 50 and 80% coverage rate baselines.

## Results

### Model fits

**Fig. 2:**
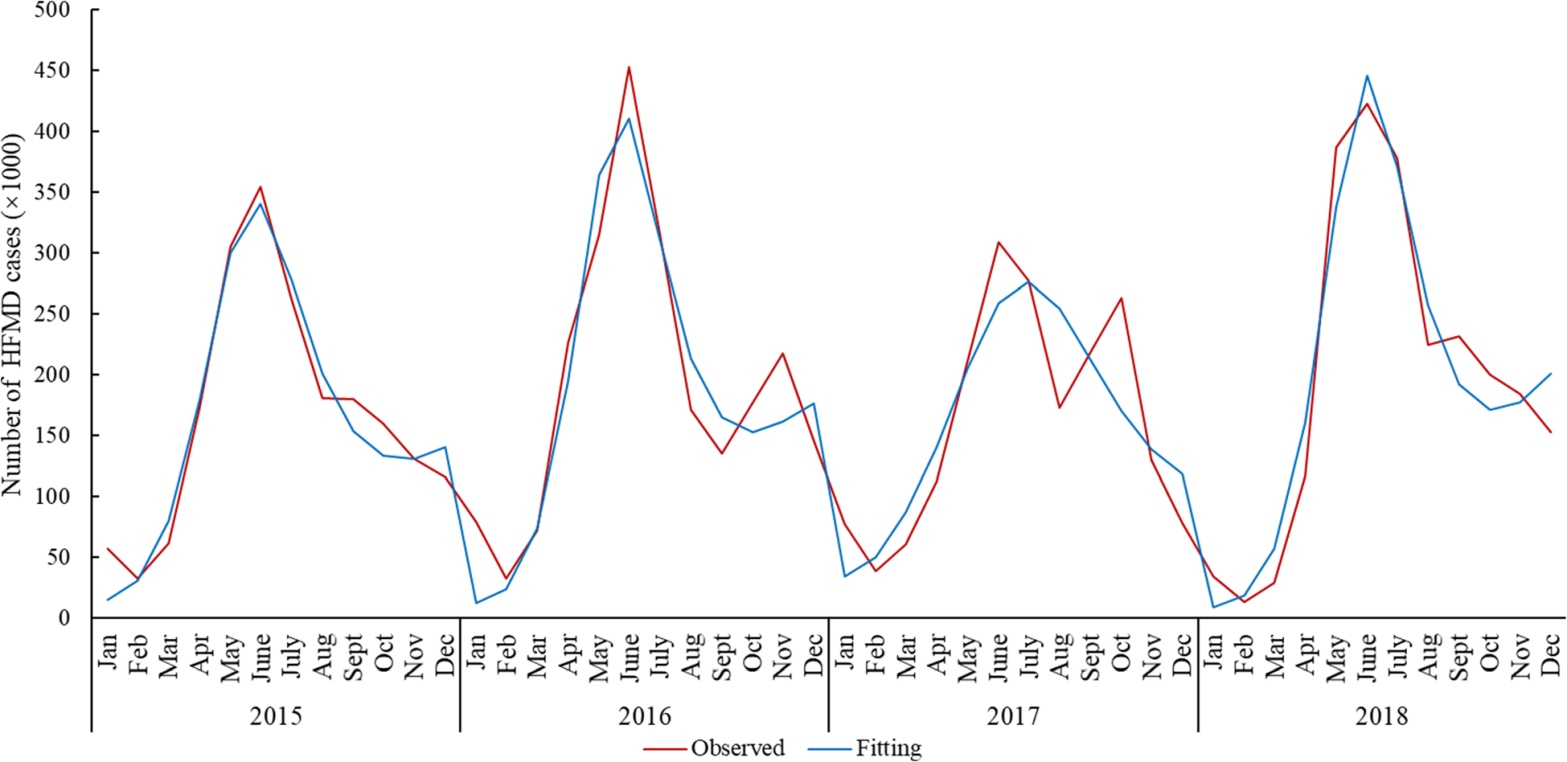
Fitting result between the simulated number and the monthly reported cases of hand, foot and mouth disease in China, 2015–2018. The observed number of HFMD cases comes from official infectious disease report from China CDC, the fitting number of HFMD cases is the sum of I_2_ and I_3_, where I_2_ indicates the number of no EV71 or CVA16 HFMD $\Big({\mathrm{I}}_{\mathrm{h}}^{-\mathrm{ec}}$) / no EV71 HFMD $\left({\mathrm{I}}_{\mathrm{h}}^{-\mathrm{e}}\right)$, I_3_ indicates the number of EV71 or CVA16 HFMD $\left({\mathrm{I}}_{\mathrm{h}}^{\mathrm{ec}}\right)$ / only EV71- HFMD $\left({\mathrm{I}}_{\mathrm{h}}^{\mathrm{e}}\right)$

The model estimates diagnosed HFMD cases in China for 2015 (*n* = 1,982,819), 2016 (*n* = 2,258,846), 2017 (n = 1,948,522) and 2018 (n = 2,398,566), which closely approximates the observed data (Fig. 2). The monthly fitting and observed results are shown in Supplementary Table 1. The model provides an adequate fit to the observed data as shown in Table 2. All the *p* values are greater than 0.05, therefore, we cannot reject the null hypothesis at the 5% significant level. The modeled result is a good estimate of the observed value. The model also captures the seasonality of disease incidence with two peaks of HFMD incidences in a given year: March to May and September to October (Fig. 2).

**Table 2:** Chi-square test results of fitting model, 2015–2018

	2015	2016	2017	2018
Degrees of freedom	3	3	3	3
Accepting value at 5% significant level	7.815	7.815	7.815	7.815
*χ2*	0.490	4.235	0.928	1.53
*P* value	> 0.05	> 0.05	> 0.05	> 0.05

### Sensitivity analysis

**Fig. 3:**
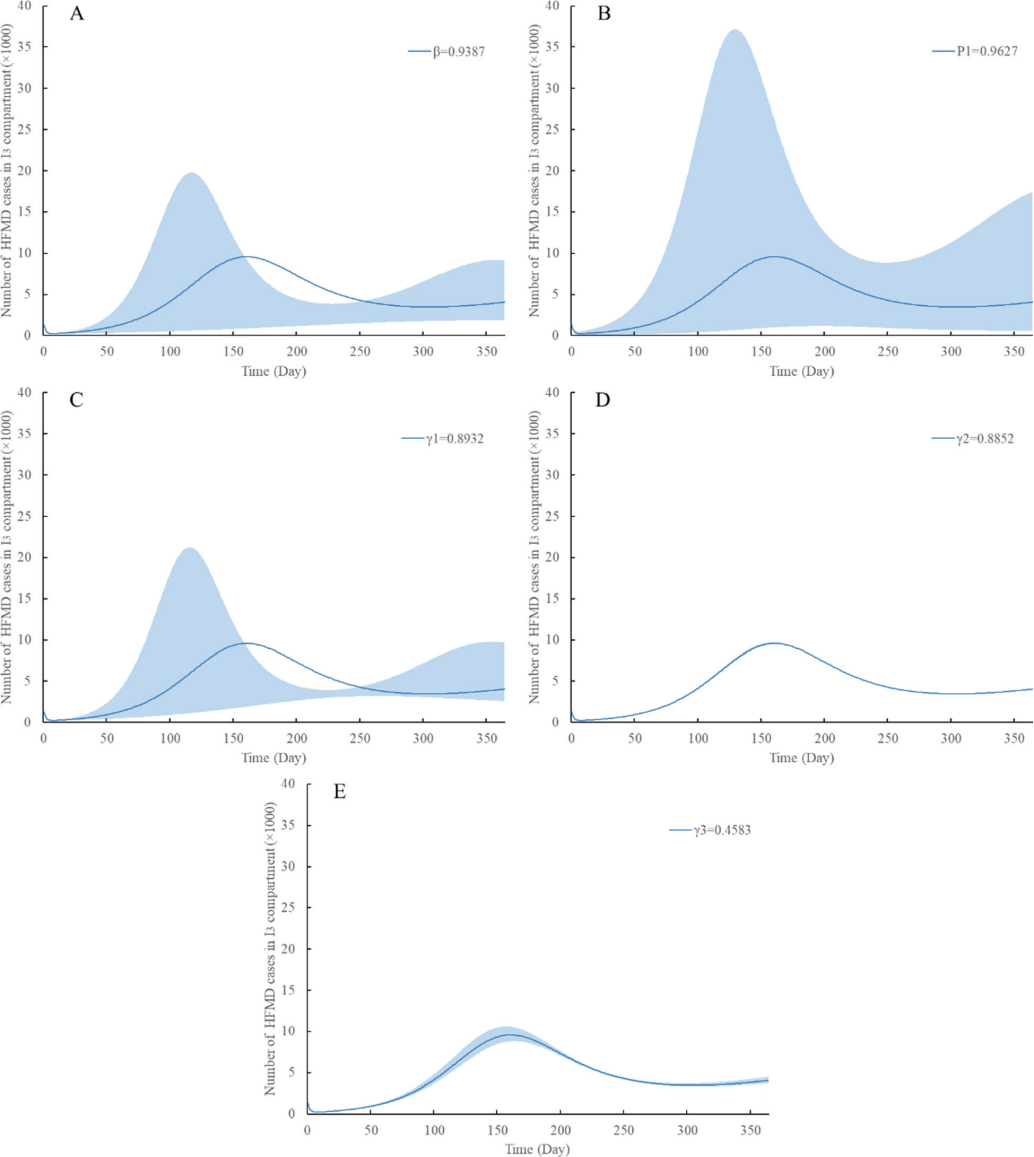
Estimated impacts of 5 parameters on the number of EV71 or CV-A16 HFMD cases (I_3_), with ranges based on sensitivity analysis. **A**, **B**, **C**, **D** and **E**: The five parameters (β, P_1_, γ_1_, γ_2_, γ_3_) range from 5% below to above of their fitting values, respectively

As we can see from the formula (4), the value of $\frac{d{R}_0}{d\beta}$ is greater than 0 and the values of $\frac{d{R}_0}{d\gamma 1}$, $\frac{d{R}_0}{d\gamma 2}$ and $\frac{d{R}_0}{d\gamma 3}$ are less than 0. But whether the value of $\frac{d{R}_0}{d{P}_1}$ is greater than 0 remains uncertain. These five parameters all range from 5% below to above of their fitting values. The influence of of β, P_1_ and γ_1_ on the number of cases are large, while the influence of γ_2_ and γ_3_ are minimal (Fig. 3).

### Impact of different vaccination type and coverage

**Fig. 4:**
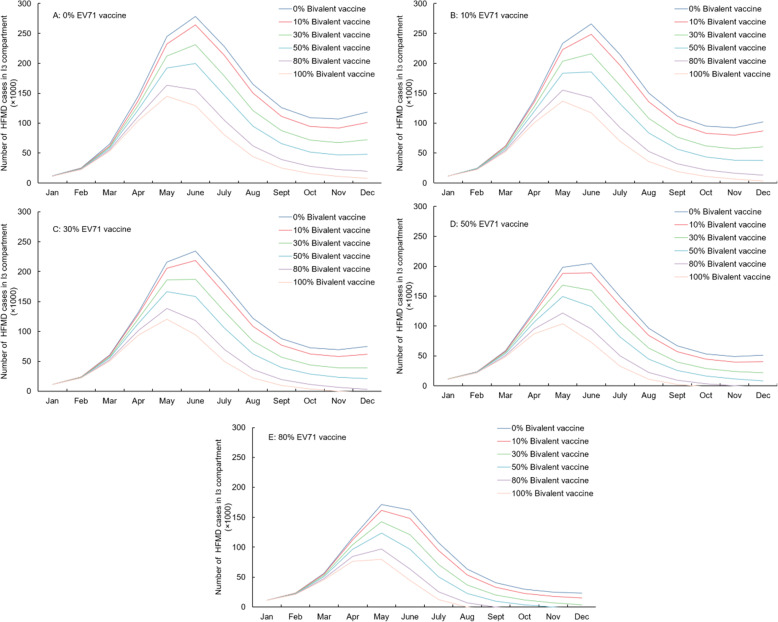
Number of HFMD cases in I_3_ changes under different EV71 and bivalent vaccine coverage rates. **A**-**E**: 0, 10, 30, 50, 80% EV71 vaccine coverage combined with 0, 10, 30, 50, 80, 100% Bivalent vaccine coverage, respectively. I_3_ indicates the number of EV71 or CVA16 HFMD $\left({\mathrm{I}}_{\mathrm{h}}^{\mathrm{ec}}\right)$ / only EV71- HFMD $\left({\mathrm{I}}_{\mathrm{h}}^{\mathrm{e}}\right)$, based on different types of vaccines

**Fig. 5:**
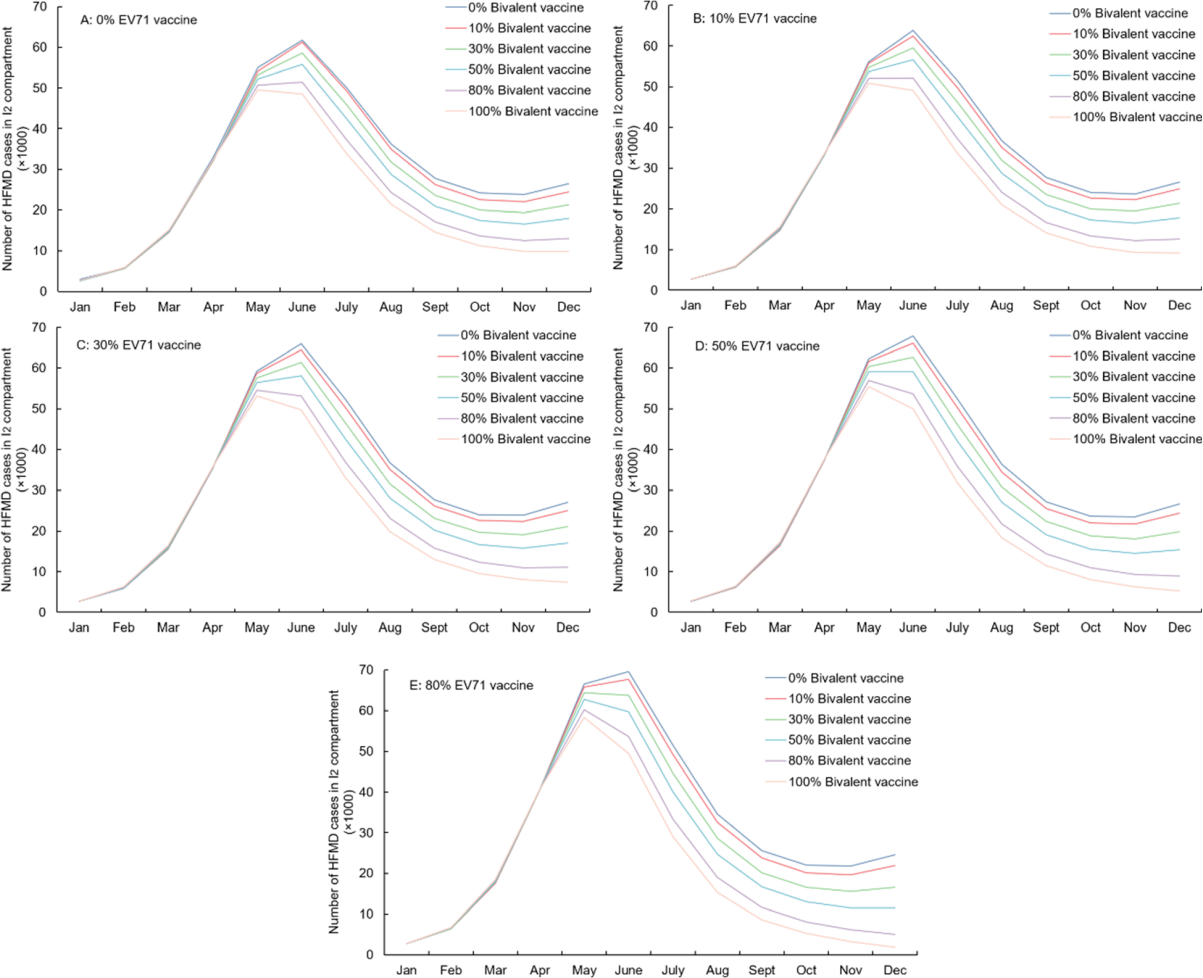
Number of HFMD cases in I_2_ changes under different EV71 and bivalent vaccine coverage rates. **A**-**E**: 0, 10, 30, 50, 80% EV71 vaccine coverage combined with 0, 10, 30, 50, 80, 100% Bivalent vaccine coverage, respectively. I_2_ indicates the number of no EV71 or CVA16 HFMD $\Big({\mathrm{I}}_{\mathrm{h}}^{-\mathrm{ec}}$) / no EV71 HFMD $\left({\mathrm{I}}_{\mathrm{h}}^{-\mathrm{e}}\right)$, based on different types of vaccine

Figures 4 and 5 and Table 3 show the impact of bivalent and monovalent vaccines on the incidence of HFMD in one year. Higher vaccine coverage led to reduced incidence but the efficiency also declined as the NNV (number needed to be vaccinated per case reduction) increased. Increased coverage of the monovalent vaccine from 0 to 80% decreased the number of cases by 797,262(49.1%) in one year. Use of a bivalent vaccine at an 80% coverage level decreased the number of HFMD cases by 828,560(51.0%) in 1 y. Figure 4 shows that when both the EV71 vaccine and the bivalent vaccine reach high coverage, incidence may be fall to zero. As shown in Figs. 4 and 5, a change in bivalent vaccine coverage has a greater impact on I_3_ than on I_2_ as expected because I_3_ reflects the number of cases of HFMD due to both serotypes. Besides, vaccine coverage also has a slight influence on the number of I_2_ (Fig. 5). This may indicate that bivalent vaccines are more effective than the sum of their individual effects.

**Table 3:** Total number of HFMD case reduction in I_3_* under different coverage scenarios over 1 year

Bivalent vaccine coverage	EV-71 vaccine coverage									
	0%		10%		30%		50%		80%	
	Case reduction (%**)	NNV***	Case reduction (%)	NNV	Case reduction (%)	NNV	Case reduction (%)	NNV	Case reduction (%)	NNV
0%	0(0.0)	–	123,776(7.6)	77.6	339,672(20.9)	84.8	537,032(33.0)	89.4	797,262(49.1)	96.3
10%	130,639(8.0)	73.5	240,476(14.8)	79.8	446,345(27.5)	86.0	633,389(39.0)	90.9	877,700(54.0)	98.4
30%	357,871(22.0)	80.5	457,647(28.2)	83.9	642,969(39.6)	89.6	808,968(49.8)	94.9	1,021,084(62.8)	103.4
50%	563,164(34.7)	85.2	652,564(40.2)	88.3	816,793(50.3)	94.0	961,374(59.2)	99.9	1,141,319(70.2)	109.3
80%	828,560(51.0)	92.7	901,989(55.5)	95.8	1,034,085(63.6)	102.1	1,146,680(70.6)	108.8	1,280,602(78.8)	119.9
100%	976,602(60.1)	98.3	1,039,339(63.9)	101.6	1,150,320(70.8)	108.5	1,242,653(76.5)	115.9	1,349,720(83.0)	128.0

*I_3_ indicates the number of EV71 or CVA16 HFMD $\left({\mathrm{I}}_{\mathrm{h}}^{\mathrm{ec}}\right)$ / only EV71- HFMD $\left({\mathrm{I}}_{\mathrm{h}}^{\mathrm{e}}\right)$, based on different types of vaccines.

**%: The percentage of the number of HFMD case reduction in I_3_ based on the initial number with no intervention

***NNV The number of persons needed to be vaccinated per case reduction

### Impact of pulse vaccination strategies

**Fig. 6:**
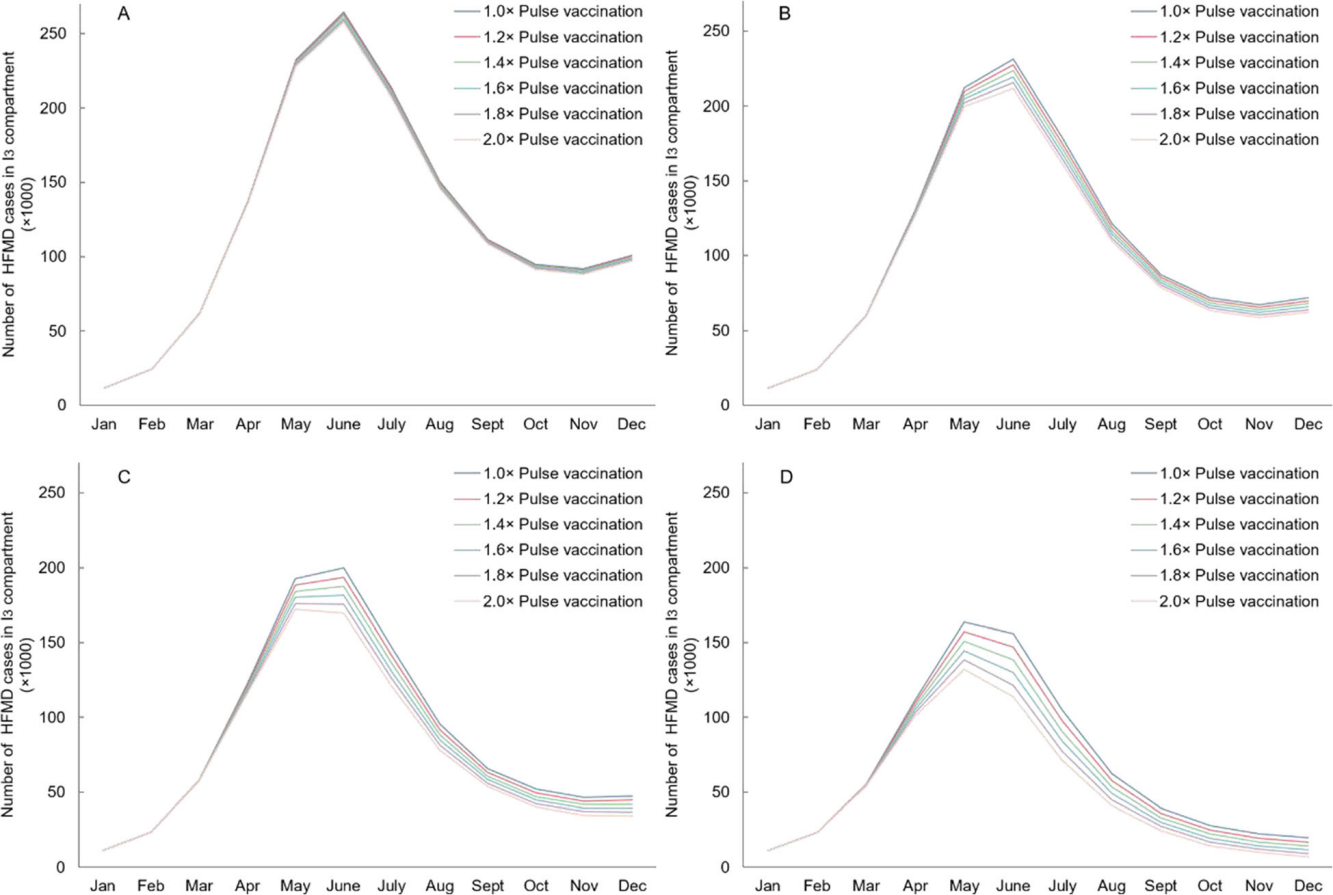
Changes of EV71 or CV-A16 HFMD (I_3_) cases under different pulse vaccination scenarios. **A**, **B**, **C**, **D**: 6 pulse intensities (1.0×, 1.2×, 1.4×, 1.6×, 1.8×, 2.0×) based on 4 different vaccination rates (26,300 person/day, 78,900 person/day, 131,500 person/day and 210,400 person/day), respectively

Figure 6 and Table 4 show the impact of different strategies of pulse vaccination on the incidence of HFMD. With baseline 10% coverage rate (vaccination rate is 26,300 person/day), increasing pulse vaccination intensity from 1.0 time to 2.0 times would increase the impact from 130,639 to 167,660 cases prevented, and the NNV would decrease from 73.5 to 71.9. Incidence consistently declined with increasing pulse vaccination intensity across different baseline coverage of the bivalent vaccine. However, the value of NNV showed a slight increase from 92.7 to 93.9 at high baseline coverage of 80%, which is the opposite of other coverage scenarios.

**Table 4:** Numbers of EV71 or CV-A16 HFMD case reduction under different pulse vaccination scenarios over 1 year

Vaccination rate (person/day)	Different pulse intensities											
	1.0×		1.2×		1.4×		1.6×		1.8×		2.0×	
	Case reduction(%*)	NNV**	Case reduction(%)	NNV	Case reduction(%)	NNV	Case reduction(%)	NNV	Case reduction(%)	NNV	Case reduction(%)	NNV
26,300	130,639(8.0)	73.5	138,076(8.5)	73.0	145,497(9.0)	72.6	152,901(9.4)	72.2	160,288(9.9)	71.9	167,660(10.3)	71.6
78,900	357,871(22.0)	80.5	378,317(23.3)	79.9	398,607(24.5)	79.5	418,742(25.8)	79.1	438,719(27.0)	78.8	458,540(28.2)	78.5
131,500	563,164(34.7)	85.2	593,997(36.5)	84.8	624,375(38.4)	84.6	654,296(40.3)	84.4	683,755(42.1)	84.2	712,750(43.9)	84.2
210,400	828,560(51.0)	92.7	869,774(53.5)	92.7	909,752(56.0)	92.9	948,482(58.4)	93.1	985,953(60.7)	93.5	1,022,152(62.9)	93.9

*%: The percentage of the EV71 or CV-A16 HFMD case reduction based on the initial number with no intervention.

**NNV: The number of persons needed to be vaccinated per case reduction.

## Discussion

There have been many papers on how to control and prevent HFMD from public health or statistical model perspectives. However, there is few work based on mathematical models to simulate dynamics of HFMD and to predict various burdens of HFMD under different scenarios of bivalent vaccine coverage. Our model extends prior work by including vaccination and reproduced the seasonal pattern of HFMD observed among children under 5 years old in China. The findings have important implications for our understanding of the spread of HFMD in China, and consideration of vaccination as a control measure to reduce the spread of HFMD.

*R*_*0*_ is an important indicator of disease outbreak. Its value indicates the number of newly infected persons during one infectious period after one infected person is introduced to an totally susceptible population. If *R*_*0*_ is larger than 1, the outbreak will take place. If *R*_*0*_ is under 1, infection will die out. In our study, the formula of *R*_*0*_ consists of four parts. This is consistent with CC Lai’s research [24]. The values of *R*_*0*_ in China from 2015 to 2018 are all greater than 1. Theoretically it is already over the threshold value for HFMD outbreaks in this period if the virus is introduced and transmitted among the children. There are other studies about the basic reproduction numbers of HFMD, and its numerical range is 1.014–1.742. Wang et al. [25] added the density of pathogen in the contaminated environments as a compartment in their model and they calculated the basic reproduction number R_0_ = 1.7424. Li et al. [26] constructed a two-stage structured model to fit the HFMD data from 2009 to 2014 in China and used the same way with us to estimate the basic reproduction number R0. In their study, R_0_ fluctuated in the range of 1.06–1.57 in 2019–2014, which is similar to our estimated range.

We also performed a sensitivity analysis to study the effects of different parameters on the incidence of HFMD. Derivative results show that *R*_*0*_ increases as β increases and γ_1_, γ_2_, γ_3_ decrease. Moreover, it’s difficult to figure out whether the recovery time courses for people who are hospitalized (1/γ_2_ and 1/γ_3_) are longer than that of people who are not hospitalized (1/γ_1_). For one, people who are hospitalized may receive effective medical treatment which would shorten the recovery time. For another, people usually go to hospital because of the severity of the condition, which means they may need more time to recover. Therefore, the value of $\frac{d{R}_0}{d{P}_1}$ depends on medical interventions and disease conditions. Considering the biological meanings of these parameters, the trend of *R*_*0*_ change is consistent with reality. More specifically, the sensitivity analysis curve shows that these five parameters (β, P_1_, γ_1_, γ_2_, γ_3_) influence the incidence of EV71 and CV-A16 HFMD in different directions, to varying degrees. Considering the biological meanings of β and γ_1_, β, also known as the transmission rate, is composed of two parts: Contact frequency × Infection efficiency. Infection efficiency is determined by the characteristics of different pathogens themselves, and it is difficult for us to intervene. However, we can change the value of contact frequency in a variety of ways. γ_1_ indicates the recovery rate of people who are infectious but not hospitalized, which is equal to the reciprocal of recovery time. Most HFMD is a self-limited disease. Therefore, this part of patients who are infectious but not hospitalized accounts for the vast majority of all HFMD patients. Considering that they don’t go to hospital, home care and self-purchasing antiviral drugs are key factors influencing the value of γ_1_ that we can intervene. Inspiringly, this provides us some new ideas to control HFMD, such as isolation of patients in the early stage of dissemination, more frequent hand-washing and ventilation [27] to lower the β value, or better treatment conditions to increase the γ1, γ2 and γ3 value.

Apart from constructing the SEIR - VEIR model to fit the reported HFMD data, we introduced vaccination to predict how HFMD incidence changes along with different vaccine coverage rates. It is generally believed that EV71 vaccine can protect against EV71 but not against CVA16 infection, which is also a major cause of HFMD [28]. Therefore, the monovalent vaccine may be less acceptable as a long-term public health strategy to control HFMD. Our findings suggest that bivalent vaccines may be the best strategy to reduce HFMD incidence. Currently, the bivalent vaccine for EV71 and CV-A16 HFMD is under development, the bivalent vaccine will be available in the nearly future. However, the number needed to be vaccinated (NNV) per case reduction suggests that the efficiency of vaccination is low. There are some possible reasons including the low probability of suffering from HFMD among whole population, which is lower than 0.2%. Moreover, the incidence rate in susceptible population (children under 5 years old) is only 2.5% [2]. Secondly, we underestimated the effectiveness of vaccines. Diseases caused by EV71 and CV-A16 are not only HFMD. We did not consider the vaccine protection effect against other diseases.

The pulse vaccination strategy consists of repeated application of vaccine at discrete time with equal interval in a population in contrast to the traditional constant vaccination [20, 29]. Pulse vaccination is considered to be a good complement to routine vaccination. Liu [16] integrated the pulse vaccination into a tuberculosis model to vaccinate the susceptible individuals periodically to overcome the shortage of the constant vaccination strategy and the effect of mixed vaccination is obvious, where the proportion of infectious individuals quickly falls down to a very low level. In our study, we introduced pulse vaccination strategy with 6 different intensities, in 4 different baselines in three specific months in view of the apparent seasonal variation of HFMD. There are two peaks of HFMD incidences in one year: May to July and September to October. From the results, we can see the decrease in NNV (number needed to be vaccinated per case reduction) value in 10, 30, and 50% coverage rates along with the pulse intensity increasing, which means as the pulse intensity increases, the efficiency of vaccine also rises up. But in 80% coverage baseline, we find the opposite result, possibly due to the inefficiency of excessive coverage itself.

Although it is somewhat unsatisfying that our analysis could not pinpoint. First, national HFMD epidemic information is calculated monthly. We cannot obtain the daily data which may help us improve the accuracy of the model. Second, serotype replacement indeed exists as the proportions of different HFMD serotypes having been changing in recent years [18], but our model in current study does not account for this transition. We assume that the proportions of different serotypes of HFMD are constant from 2015 to 2018, with reference to baseline levels [19]. Therefore, this may lead to differences from the real HFMD epidemic. Third, EV71 vaccine was launched in China at the end of 2016. But during our investigation, we find people’s acceptance of this vaccine is generally low due to its limited effectiveness and its high price. Therefore, some results are just based on our hypothesis but may not be realized in the future considering the cost-effectiveness. Apart from these, we conducted a simple pulse vaccination analysis and just introduced 6 pulse intensities in 3 specific months. However, we have no idea about what intensity of the pulse or pulse vaccination in which months can achieve the maximum of cost-effectiveness.

Overall, as HFMD can be caused by numerous serotypes of enteroviruses, the existing EV71 vaccine has no cross-protection against other subtypes. The epidemic of HFMD is still developing in China. Although EV71 vaccine provides us a more specific way to control the HFMD caused by EV71, which is the leading cause of severe and fatal HFMD, its preventive effect and cost-effectiveness on the overall HFMD epidemic is very limited. Other researchers have found that the proportion of EV71-caused HFMD decreased with the popularization of EV71 vaccination, while the proportion of HFMD caused by other serotypes such as CV-A16 and CV-A10 increased [18]. This suggests multivalent vaccine and more effective vaccination strategy different from routine inoculation, such as pulse vaccination, would play an important role in preventing the transmission of all-type-HFMD in the future.

## Supplementary Information

**Additional file 1: Table S1.** The monthly fitting and observed cases of hand, foot and mouth disease in China from 2015 to 2018.

**Additional file 2**

## Data Availability

The datasets used and/or analysed during the current study are available from the corresponding author on reasonable request.
