# Supplementary material for: The burden of hand, foot, and mouth disease among children under different vaccination scenarios in China: a dynamic modelling study
Source: BMC Infect Dis. 2021 Jul 5;21:650. doi: 10.1186/s12879-021-06157-w (PMC8259139; doi:10.1186/s12879-021-06157-w)
Supplement: Supplementary file 1 — Additional file 1: Table S1. The monthly fitting and observed cases of hand, foot and mouth disease in China from 2015 to 2018. [file 12879_2021_6157_MOESM1_ESM.docx]

The burden of hand, foot, and mouth disease among children under different vaccination scenarios in China: a dynamic modelling study

Zhixi Liu^1†^, Jie Tian^1†^, Yue Wang^1^, Yixuan Li^1^, Jing Liu-Helmersson^2^, Sharmistha Mishra^3,4^, Abram L. Wagner^5^, Yihan Lu^1^, Weibing Wang^1*^

^1^School of Public Health, Fudan University, Shanghai 200032, China

^2^Department of Epidemiology and Global Health, Faculty of Medicine, Umeå University, Umeå 90187, Sweden

^3^Department of Medicine, Institute of Medical Sciences, and Institute of Health Policy, Management and Evaluation, University of Toronto, Canada

^4^Li Ka Shing Knowledge Institute, Center for Urban Health Solutions, University of Toronto, Canada

^5^Department of Epidemiology, University of Michigan, Ann Arbor, MI, 48109, USA

* Corresponding authors:

Dr. Weibing Wang

School of Public Health, Fudan University, Shanghai 200032, China

Email: wwb@fudan.edu.cn

Table S1. The monthly fitting and observed cases of hand, foot and mouth disease in China from 2015 to 2018

| Month | 2015 | |  | 2016 | |  | 2017 | |  | 2018 | |  |
| --- | --- | --- | --- | --- | --- | --- | --- | --- | --- | --- | --- | --- |
|  | Observed | Fitting |  | Observed | Fitting |  | Observed | Fitting |  | Observed | Fitting |  |
| Jan | 57,763 | 15,178 |  | 79,499 | 12,886 |  | 77,412 | 34,247 |  | 34,800 | 8,861 |  |
| Feb | 33,003 | 30,827 |  | 32,457 | 23,746 |  | 38,654 | 50,247 |  | 13,306 | 18,610 |  |
| Mar | 61,378 | 79,961 |  | 72,464 | 73,538 |  | 61,225 | 86,824 |  | 28,856 | 57,684 |  |
| Apr | 173,384 | 178,825 |  | 226,430 | 194,889 |  | 112,898 | 140,727 |  | 116,941 | 160,383 |  |
| May | 304,863 | 300,023 |  | 315,077 | 364,338 |  | 211,189 | 205,403 |  | 387,135 | 338,056 |  |
| June | 354,308 | 340,114 |  | 452,668 | 410,325 |  | 308,789 | 259,105 |  | 423,018 | 445,125 |  |
| July | 262,239 | 278,093 |  | 315,077 | 310,129 |  | 277,680 | 276,731 |  | 377,629 | 371,382 |  |
| Aug | 180,813 | 201,230 |  | 171,581 | 213,074 |  | 173,367 | 254,603 |  | 225,058 | 257,343 |  |
| Sept | 180,074 | 153,742 |  | 134,972 | 165,226 |  | 218,880 | 212,305 |  | 231,775 | 192,401 |  |
| Oct | 160,106 | 133,351 |  | 177,639 | 152,495 |  | 263,311 | 170,611 |  | 199,938 | 170,947 |  |
| Nov | 130,829 | 130,995 |  | 217,754 | 161,440 |  | 130,280 | 139,037 |  | 184,396 | 177,126 |  |
| Dec | 116,239 | 140,480 |  | 145,558 | 176,760 |  | 78,750 | 118,681 |  | 153,086 | 200,647 |  |
| Total | 2,014,999 | 1,982,819 |  | 2,341,176 | 2,258,846 |  | 1,952,435 | 1,948,521 |  | 2,375,938 | 2,398,566 |  |
