# Supplementary material for: The burden of hand, foot, and mouth disease among children under different vaccination scenarios in China: a dynamic modelling study
Source: BMC Infect Dis. 2021 Jul 5;21:650. doi: 10.1186/s12879-021-06157-w (PMC8259139; doi:10.1186/s12879-021-06157-w)
Supplement: Supplementary file 2 — Additional file 2 [file 12879_2021_6157_MOESM2_ESM.docx]

The burden of hand, foot, and mouth disease among children under different vaccination scenarios in China: a dynamic modelling study

Zhixi Liu^1†^, Jie Tian^1†^, Yue Wang^1^, Yixuan Li^1^, Jing Liu-Helmersson^2^, Sharmistha Mishra^3,4^, Abram L. Wagner^5^, Yihan Lu^1^, Weibing Wang^1*^

^1^School of Public Health, Fudan University, Shanghai 200032, China

^2^Department of Epidemiology and Global Health, Faculty of Medicine, Umeå University, Umeå 90187, Sweden

^3^Department of Medicine, Institute of Medical Sciences, and Institute of Health Policy, Management and Evaluation, University of Toronto, Canada

^4^Li Ka Shing Knowledge Institute, Center for Urban Health Solutions, University of Toronto, Canada

^5^Department of Epidemiology, University of Michigan, Ann Arbor, MI, 48109, USA

* Corresponding authors:

Dr. Weibing Wang

School of Public Health, Fudan University, Shanghai 200032, China

Email: wwb@fudan.edu.cn

**R_0_ calculation process**

**1.** The next-generation matrix approach in [1] is applied to calculate the basic reproduction ratio, R_0_. Let $F_{i}\left( x \right)$ be the rate of appearance of new infections in compartment *i*, $V_{i}^{+}\left( x \right)$ be the rate of transfer of individuals into compartment *i* by all other means, and $V_{i}^{-}\left( x \right)$ be the rate of transfer of individuals out of compartment *i*. The disease transmission model consists of non-negative initial conditions together with the following system of equations: $x_{i}=f_{i}(x)= F_{i}\left( x \right)-V_{i}\left( x \right)$, where $V_{i}=V_{i}^{-}-V_{i}^{+}$.The entry of the product $FV^{-1}$ is the expected number of new infections in compartment I produced by the infected individual originally introduced into compartment k. $FV^{-1}$ is the next generation matrix for the model and $R_{0}=\rho\left（ FV^{-1} \right）$, where *ρ(A)* denotes the spectral radius of a matrix A.

**2. the dynamics of HFMD without vaccination.**

$\left\{ \begin{aligned} & \frac{dS}{dt}=\pi N+\gamma R-\frac{\beta S\sum I}{N}-\mu S \\ & \frac{dE_{s}}{dt}=\frac{\beta S\sum I}{N}-\alpha(P_{1}{+P}_{2}+P_{3})E_{s}-\mu E_{s} \\ & \frac{dI_{N}}{dt}=\alpha P_{1}E_{s}-\gamma_{1}I_{N}-\mu I_{N} \\ & \frac{dI_{2}}{dt}=\alpha P_{2}E_{s}-\gamma_{2}I_{2}-\mu I_{2} \\ & \frac{dI_{3}}{dt}=\alpha P_{3}E_{S}-\gamma_{3}I_{3}-\mu I_{3} \\ & \frac{dR}{dt}=\gamma_{1}I_{N}+\gamma_{2}I_{2}+\gamma_{3}I_{3}-\gamma R-\mu R \end{aligned} \right.$ (1)

We can write the right-hand side of model (1) as *f-v* with

$f=\left( \begin{matrix} \frac{\beta S\sum I}{N} \\ 0 \\ 0 \\ 0 \end{matrix} \right)$,

$v=\left( \begin{matrix} \alpha(P_{1}{+P}_{2}+P_{3})E_{s}+\mu E_{s} \\ -\alpha P_{1}E_{s}+\gamma_{1}I_{N}+\mu I_{N} \\ -\alpha P_{2}E_{s}+\gamma_{2}I_{2}+\mu I_{2} \\ -\alpha P_{3}E_{S}+\gamma_{3}I_{3}+\mu I_{3} \end{matrix} \right)$.

Calculating the Jacobian matrices, *F* and *V*, at the DFE, we have

$F=\left[ \begin{matrix} \begin{matrix} 0 & \beta\\ 0 & 0 \end{matrix} & \begin{matrix} \beta& \beta\\ 0 & 0 \end{matrix} \\ \begin{matrix} 0 & 0 \\ 0 & 0 \end{matrix} & \begin{matrix} 0 & 0 \\ 0 & 0 \end{matrix} \end{matrix} \right]$,

$V=\left[ \begin{matrix} \alpha\left( P_{1}{+P}_{2}+P_{3} \right)+\mu& 0 & 0 & 0 \\ -\alpha P_{1} & \gamma_{1}+\mu& 0 & 0 \\ -\alpha P_{2} & 0 & \gamma_{2}+\mu& 0 \\ -\alpha P_{3} & 0 & 0 & \gamma_{3}+\mu\end{matrix} \right]$.

The basic reproduction number *R_0_* is the spectral radius of *FV^-1^*. Here we use P_1_ to represent P_2_ and P_3_ ($P_{2}=0.3\times(1-P_{1})$, $P_{3}=0.7\times(1-P_{1})$) and the result is as follow:

$R_{0}^{2015,2016}=\frac{\alpha\beta}{\alpha+\mu}\left( \frac{P_{1}}{\mu+\gamma_{1}}+\frac{0.3\text{ }-0.3P_{1}}{\mu+\gamma_{2}}+\frac{0.7\text{ }-0.7P_{1}}{\mu+\gamma_{3}} \right)$ (2)

Substituting the parameter values into the formula (2) can get *R_0_*=1.08 in 2015 and *R_0_*=1.10 in 2016.

**3. the dynamics of HFMD with vaccination**

$\left\{ \begin{aligned} &\frac{dS}{dt}=\pi N+\gamma R-\frac{\beta S\sum I}{N}-v-\mu S \\ &\frac{dV}{dt}=v-\frac{\beta V\sum I}{N}-\mu V \\ &\frac{dE_{s}}{dt}=\frac{\beta S\sum I}{N}-\alpha(P_{1}{+P}_{2}+P_{3})E_{s}-\mu E_{s} \\ &\frac{dE_{v}}{dt}=\frac{\beta V\sum I}{N}-\alpha\times\left[ P_{1}+P_{2}+\left( 1-VE \right)\times P_{3} \right]E_{v}-\mu E_{v} \\ &\frac{dI_{N}}{dt}=\alpha P_{1}{(E}_{S}+E_{v})-\gamma_{1}I_{N}-\mu I_{N} \\ &\frac{dI_{2}}{dt}=\alpha P_{2}{(E}_{S}+E_{v})-\gamma_{2}I_{2}-\mu I_{2} \\ &\frac{dI_{3}}{dt}=\alpha P_{3}E_{S}+\left( 1-VE \right)\alpha P_{3}E_{v}-\gamma_{3}I_{3}-\mu I_{3} \\ &\frac{dR}{dt}=\gamma_{1}I_{N}+\gamma_{2}I_{2}+\gamma_{3}I_{3}-\gamma R-\mu R \end{aligned} \right.$ (3)

We can write the right-hand side of model (3) as *f-v* with

$f=\left( \begin{matrix} \frac{\beta S\sum I}{N} \\ \frac{\beta V\sum I}{N} \\ 0 \\ 0 \\ 0 \end{matrix} \right)$,

$v=\left( \begin{matrix} \alpha(P_{1}{+P}_{2}+P_{3})E_{s}+\mu E_{s} \\ \alpha\times\left[ P_{1}+P_{2}+\left( 1-VE \right)\times P_{3} \right]E_{v}+\mu E_{v} \\ -\alpha P_{1}{(E}_{S}+E_{v})+\gamma_{1}I_{N}+\mu I_{N} \\ -\alpha P_{2}{(E}_{S}+E_{v})+\gamma_{2}I_{2}+\mu I_{2} \\ -\alpha P_{3}E_{S}-\left( 1-VE \right)\alpha P_{3}E_{v}+\gamma_{3}I_{3}+\mu I_{3} \end{matrix} \right)$.

Calculating the Jacobian matrices, *F* and *V*, at the DFE, we have

$F=\left[ \begin{matrix} 0 & 0 & \beta S/N & \beta S/N & \beta S/N \\ 0 & 0 & \beta V/N & \beta V/N & \beta V/N \\ 0 & 0 & 0 & 0 & 0 \\ 0 & 0 & 0 & 0 & 0 \\ 0 & 0 & 0 & 0 & 0 \end{matrix} \right]$,

$V=\left[ \begin{matrix} \alpha\left( P_{1}{+P}_{2}+P_{3} \right)+\mu& 0 & 0 & 0 & 0 \\ 0 & \alpha\times\left[ P_{1}+P_{2}+\left( 1-VE \right)\times P_{3} \right]+\mu& 0 & 0 & 0 \\ -\alpha P_{1} & -\alpha P_{1} & \gamma_{1}+\mu& 0 & 0 \\ -\alpha P_{2} & -\alpha P_{2} & 0 & \gamma_{2}+\mu& 0 \\ -\alpha P_{3} & -\left( 1-VE \right)\alpha P_{3} & 0 & 0 & \gamma_{3}+\mu\end{matrix} \right]$.

Because the number of patients is very small, we approximate $N=S+V$. We let $c=V/N$. Then the basic reproduction number *R_0_* is the spectral radius of *FV^-1^*, Due to the large number of parameters, the formula characters are too complicated and cannot be simplified. The result is as follows:

$R_{0}^{2017,2018}= \alpha\beta\left( \alpha P_{1}^{2}\left( \gamma_{2}+\mu\right)\left( \gamma_{3}+\mu\right)+\mu\left( \gamma_{3}+\mu\right)\left( P_{2}\left( \gamma_{1}+\mu\right)+P_{1}\left( \gamma_{2}+\mu\right) \right)-P_{3}\mu\left( \gamma_{1}+\mu\right)\left( \gamma_{2}+\mu\right)\left( -1+cVE \right)+\alpha\left( \gamma_{1}+\mu\right)\left( P_{2}^{2}\left( \gamma_{3}+\mu\right)-P_{3}^{2}\left( \gamma_{2}+\mu\right)\left( -1+VE \right)+P_{2}P_{3}\left( \gamma_{2}+\gamma_{3}+2\mu-c\gamma_{2}VE-\gamma_{3}VE+c\gamma_{3}VE-\mu VE \right) \right)+\alpha P_{1}\left( P_{2}\left( \gamma_{3}+\mu\right)\left( \gamma_{1}+\gamma_{2}+2\mu\right)-P_{3}\left( \gamma_{2}+\mu\right)\left( \mu\left( -2+VE \right)+\gamma_{3}\left( -1+VE-cVE \right)+\gamma_{1}\left( -1+cVE \right) \right) \right) \right)/\left( \gamma_{1}+\mu\right)\left( \gamma_{2}+\mu\right)\left( \gamma_{3}+\mu\right)\left( \mu+\alpha\left( P_{1}+P_{2}+P_{3} \right) \right)\left( \mu+\alpha\left( P_{1}+P_{2}+P_{3}-P_{3}VE \right) \right)$ (4)

Substituting the parameter values into the formula (4) can get *R_0_*=1.36 in 2017 and *R_0_*=1.17 in 2018.

**Reference**

1. van den Driessche P, Watmough J: **Reproduction numbers and sub-threshold endemic equilibria for compartmental models of disease transmission**. *Math Biosci* 2002, **180**:29-48.
